# Transcriptional upregulation of c-MET is associated with invasion and tumor budding in colorectal cancer

**DOI:** 10.18632/oncotarget.12933

**Published:** 2016-10-26

**Authors:** Conor A. Bradley, Philip D. Dunne, Victoria Bingham, Stephen McQuaid, Hajrah Khawaja, Stephanie Craig, Jackie James, Wendy L. Moore, Darragh G. McArt, Mark Lawler, Sonali Dasgupta, Patrick G. Johnston, Sandra Van Schaeybroeck

**Affiliations:** ^1^ Drug Resistance Group, Centre for Cancer Research and Cell Biology, School of Medicine, Dentistry and Biomedical Science, Queen's University Belfast, Belfast, UK; ^2^ Tissue Pathology, Belfast Health and Social Care Trust, Belfast City Hospital, Belfast, UK

**Keywords:** c-MET, colorectal cancer (CRC), tumor budding, invasion, metastasis

## Abstract

c-MET and its ligand HGF are frequently overexpressed in colorectal cancer (CRC) and increased c-MET levels are found in CRC liver metastases. This study investigated the role of the HGF/c-MET axis in regulating migration/invasion in CRC, using pre-clinical models and clinical samples. Pre-clinically, we found marked upregulation of c-MET at both protein and mRNA levels in several invasive CRC cells. Down-regulation of c-MET using RNAi suppressed migration/invasion of parental and invasive CRC cells. Stimulation of CRC cells with rh-HGF or co-culture with HGF-expressing colonic myofibroblasts, resulted in significant increases in their migratory/invasive capacity. Importantly, HGF-induced c-MET activation promoted rapid downregulation of c-MET protein levels, while the *MET* transcript remained unaltered. Using RNA *in situ* hybridization (RNA ISH), we further showed that *MET* mRNA, but not protein levels, were significantly upregulated in tumor budding foci at the invasive front of a cohort of stage III CRC tumors (*p* < 0.001). Taken together, we show for the first time that transcriptional upregulation of *MET* is a key molecular event associated with CRC invasion and tumor budding. This data also indicates that RNA ISH, but not immunohistochemistry, provides a robust methodology to assess *MET* levels as a potential driving force of CRC tumor invasion and metastasis.

## INTRODUCTION

Colorectal cancer (CRC) is a major cause of cancer related death worldwide, with significant variation in prognosis between early and late stage disease. Five year overall survival (OS) rates for patients with localised stage II disease are as high as 80% following surgery, dropping to 40–60% for patients with stage III disease who display evidence of tumor invasion and regional lymph node metastasis [1]. Tumor budding is defined by the presence of individual tumor cells and/or small clusters of cancer cells at the invasive front of tumors [2]. Tumor budding at the invasive front has long been associated with presence of lymph node and distant metastasis, increased risk of relapse and is now a well-accepted prognostic factor in CRC [3–5]. However, the underlying biology driving the distinct prognostic differences between localised tumors and those which have developed an increased invasive capacity is, as yet, not fully understood at the molecular level.

Recent studies have highlighted the considerable intra-tumoral heterogeneity that exists within CRC primary tumors. A recent consensus molecular classification of stage II/III CRC has identified the poor prognostic CMS 4 subgroup, characterized by epithelial-mesenchymal transition (EMT) and stem-like transcriptional signatures [6]. Further interrogation of this CRC subgroup revealed the stromal-rich nature of these tumors, particularly the presence of fibroblasts, which account for the high levels of mesenchymal associated genes expressed within the CMS 4 subtype [7, 8]. These studies indicated that the prognostic value of the CMS 4 subtype is inherently associated with fibroblast infiltration, and suggested that widespread EMT does not occur within the epithelial tumor bulk. Other studies have shown that the interaction between epithelial cancer cells at the invasive front or budding cells with surrounding stromal cells or their secreted factors can locally affect Wnt/β-catenin signalling within these cells, triggering stemness, EMT and an invasive behaviour, suggesting that this pathway may be an important driver of the metastatic process and potential therapeutic target in budding cells [9–12].

The interplay between cancer associated fibroblasts (CAFs) and tumor cells, leading to metabolic reprogramming of cancer and stromal cells and activation of an EMT and invasive programme, has been described. Tumor-derived secreted factors such as TGF-β1, bFGF, and IL-6, have been shown to control the activation of cancer associated fibroblasts (CAFs), and the proteome/secretome of these CAFs, including hepatocyte growth factor (HGF), has been found to enhance the invasiveness of cancer cells [13, 14]. The prognostic and predictive value of tumor-infiltrating fibroblasts is not a new concept [15–17]. The recent tumor profiling efforts [18, 19] have also failed to identify specific epithelial-derived factors associated with the extremely small proportion of cells undergoing invasion and budding at the invasive front. In order to model CRC cell invasion/metastasis, our group has developed invasive CRC daughter cells, which display stem-like characteristics, an EMT phenotype and increased migratory/invasive levels compared to their parental counterparts [20, 21].

The receptor tyrosine kinase c-MET (mesenchymal-epithelial transition factor), encoded by the *MET* proto-oncogene, and its cognate high-affinity ligand HGF control invasive growth through the coordination of cell proliferation, survival, EMT and migration/invasion [22, 23]. c-MET expression in CRC primary tumors has been found to be predictive of local tumor invasion and regional lymph node metastasis [24] and higher c-MET levels have been found in synchronous CRC liver metastasis compared to levels obtained in matched primary tumors [25, 26]. We have previously shown that MEK1/2 inhibition-induced c-MET activation is an acute mechanism of resistance to MEK1/2 inhibitors in *RAS* and *BRAF* mutant CRC [27, 28].

In this study, we show that c-MET protein *and* mRNA levels are significantly increased in our CRC invasive models and that treatment with c-MET-specific siRNA abrogates migration/invasion of parental and invasive CRC cells. We also demonstrate that incubation of CRC cells with rh-HGF or co-culture with HGF-expressing myofibroblasts significantly increases migration/invasion and this was associated with rapid downregulation of c-MET protein but *not* mRNA levels. Using RNA *in situ* hybridization (RNA ISH) and immunohistochemistry (IHC), we show high MET mRNA but *not* protein levels in tumor budding foci at the invasive front of stage III CRC tumors. Taken together, our results indicate for the first time that elevated *MET* mRNA levels is associated with increased invasive capacity *in vitro* and the presence of tumor budding *in vivo*, suggesting that c-MET targeted therapies may represent a promising strategy to prevent invasion and disease recurrence in stage III CRC.

## RESULTS

### c-MET is a key regulator of CRC cell invasion and migration *in vitro*

**Figure 1:**
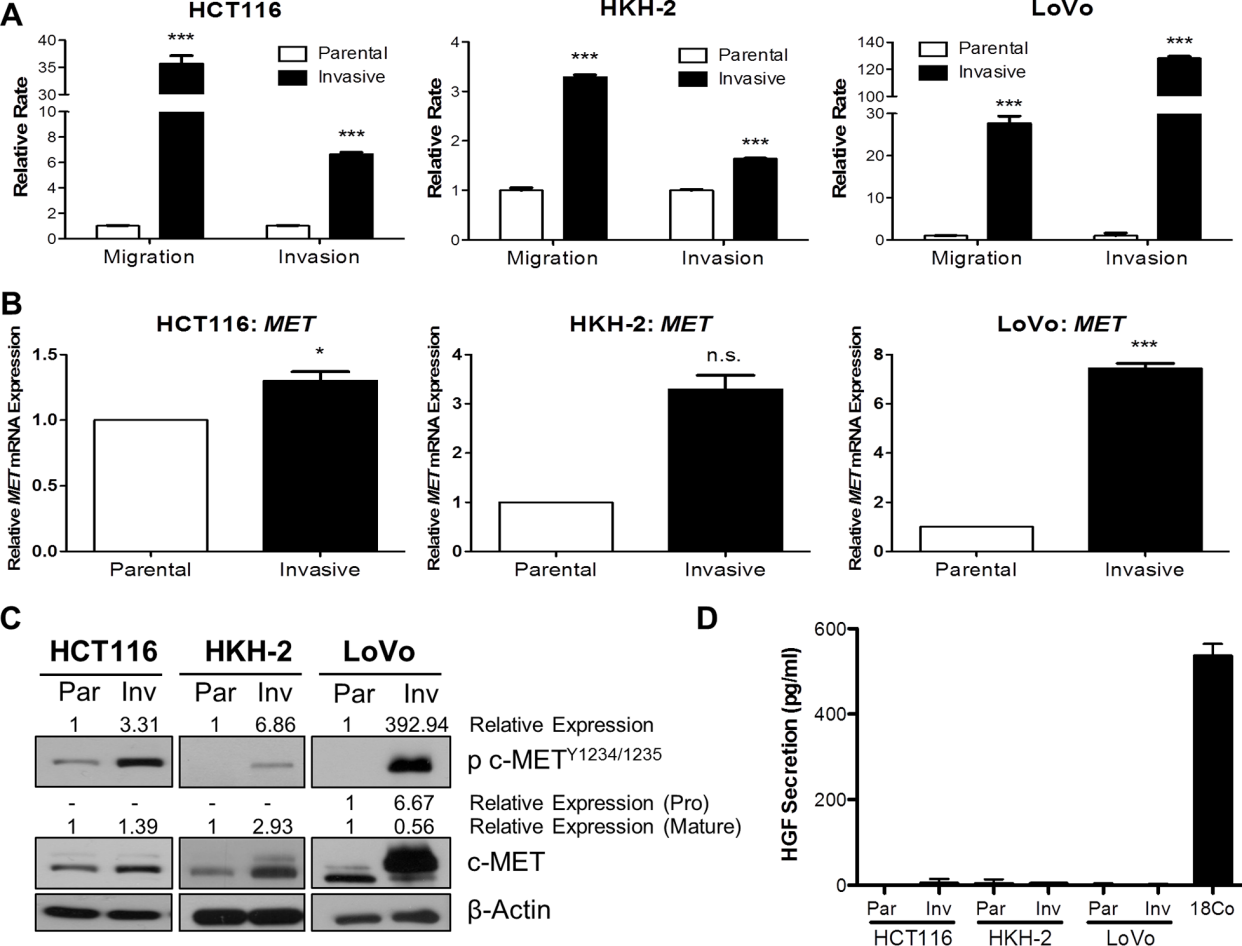
c-MET protein and mRNA levels are highly upregulated in invasive CRC daughter cell lines BD BioCoat Matrigel invasion chambers were used to isolate invasive subpopulations from a panel of CRC cells. (**A**) Migration and invasion assays of invasive subpopulations of HCT116, HKH-2 and LoVo sublines were compared with the parental cells using the quantitative xCELLigence system. (**B**) qRT-PCR assessment of *MET* gene expression levels in parental and invasive cell models. (**C**) Western blot analysis of pc-MET^1234/1235^ and c-MET protein expression levels in parental (Par) and invasive (Inv) cell models. (**D**) HGF protein levels in the culture media of parental (Par) and invasive (Inv) subpopulations were measured by ELISA [Positive control: CCD-18Co (18Co)]. *= *p* < 0.05, *** = *p* < 0.001, n.s. = not significant.

In order to identify molecular drivers of invasion and metastasis, we generated preclinical *in vitro* invasive HCT116 and HKH-2 CRC models which display an EMT-like invasive phenotype [20, 21]. Initially, we also developed invasive cell populations from the LoVo CRC cell line, using Matrigel Invasion Chambers. Using the XCELLigence real-time cell migration/invasion tracking system, we confirmed the invasion/migration rates of our invasive models, and found a 35.7-fold, 3.29-fold and 27.7-fold increase in migration rates (*P* < 0.001) and 6.65-fold, 1.64-fold and 128-fold increase in invasion rates (*P* < 0.001) in invasive HCT116, HKH-2 and LoVo cells respectively (Figure 1A). We assessed *MET* gene expression in these invasive models using qRT-PCR and found marked upregulated *MET* mRNA levels in the invasive subpopulations compared to their parental counterparts (Figure 1B). Increased *MET* mRNA levels were associated with increased c-MET protein levels in invasive subpopulations as compared to their matched parental cells (Figure 1C). Interestingly, we also observed marked increases in c-MET phosphorylation levels at kinase domain residues Y^1234/1235^ in all our invasive cell models, indicating that increased kinase activity of the receptor was concordant with c-MET overexpression (Figure 1C). Quantification of human HGF (h-HGF) protein secretion by ELISA illustrated that both parental and invasive CRC cell lines did not secrete physiologically detectable levels of h-HGF into the culture medium, indicating that overexpression and phosphorylation of c-MET in invasive cell lines is independent of autocrine-HGF secretion (Figure 1D). Consistent with this finding, we observed no detectable *HGF* mRNA levels in our CRC cell line models (Supplementary Figure S1).

**Figure 2:**
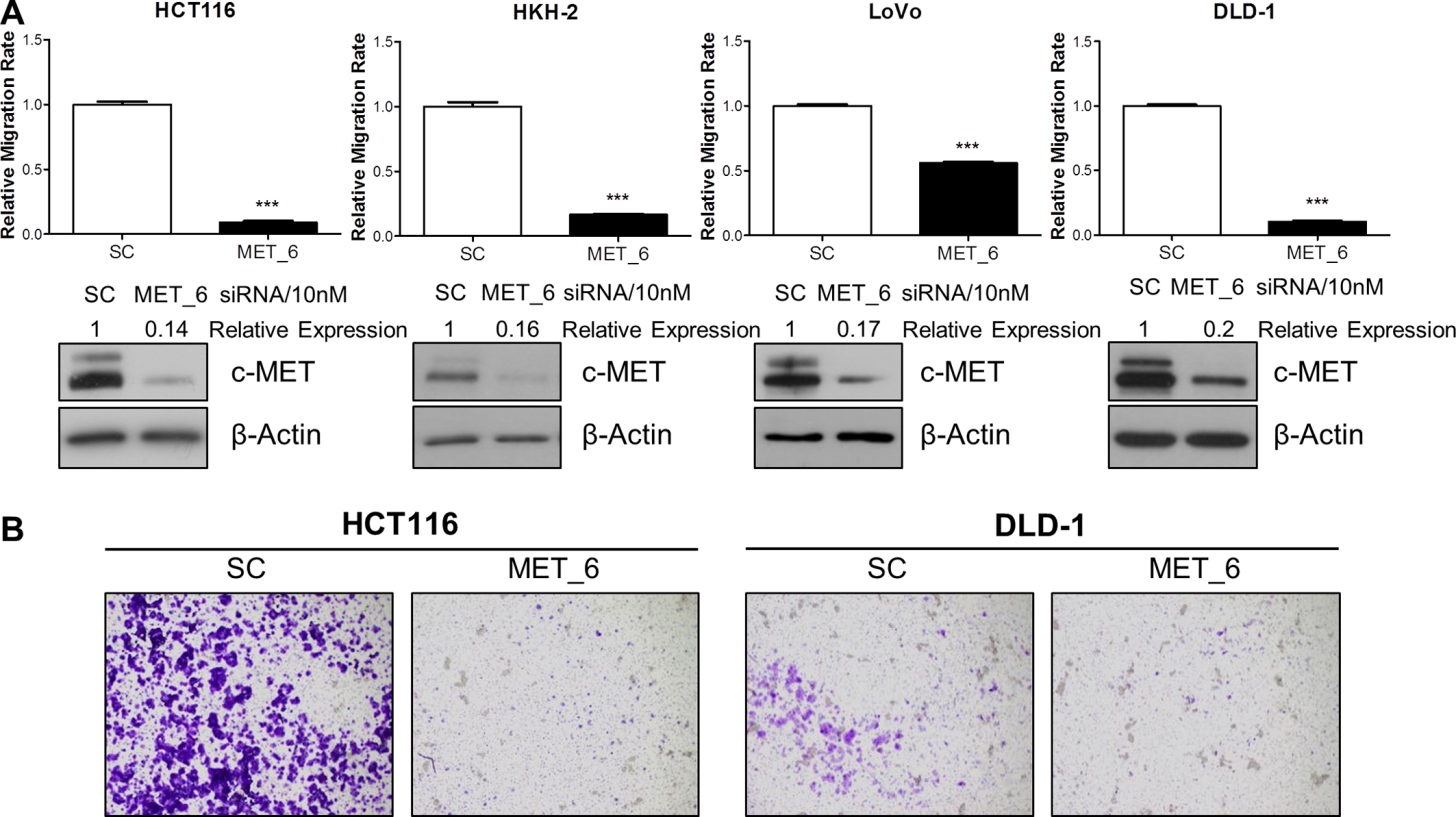
c-METi reduces migration and invasion of CRC cell lines (**A**) CRC cells were transfected with 10nM SC or 10nM c-MET siRNA (MET_6) for 24 hours and the effect on migration was determined using the xCELLigence system. Corresponding c-MET protein knockdown was confirmed by Western blotting. (**B**) Qualitative invasion rate assessment was performed following transfection with 10 nM SC or 10nM c-MET siRNA (MET_6), using Boyden chambers. ***= *p* < 0.001.

In order to investigate a potential role for c-MET in regulating migration and invasion, we employed different siRNA sequences directed against *MET* in the HCT116, HKH-2, LoVo, and DLD-1 parental and invasive CRC cells. Using the XCELLigence real-time cell migration tracking system, we found that loss of *MET* gene expression resulted in statistically significant attenuation of relative migration rate in parental and invasive cell line models, with a 44–92% reduction compared to scrambled control (SC)-treated cells (*p* < 0.001 for all cell line models) (Figure 2A and Supplementary Figure S2A). This effect was also evident using *in vitro* Boyden chamber assays, where siMET resulted in marked reduced invasion in HCT116 and DLD-1 cell lines (Figure 2B). Importantly, the decreased migration/invasion rates observed following siMET was not due to increased cell death or changes in the cell cycle profile (Supplementary Figure S2B).

### HGF-mediated activation of c-MET promotes CRC cell migration and invasion

**Figure 3:**
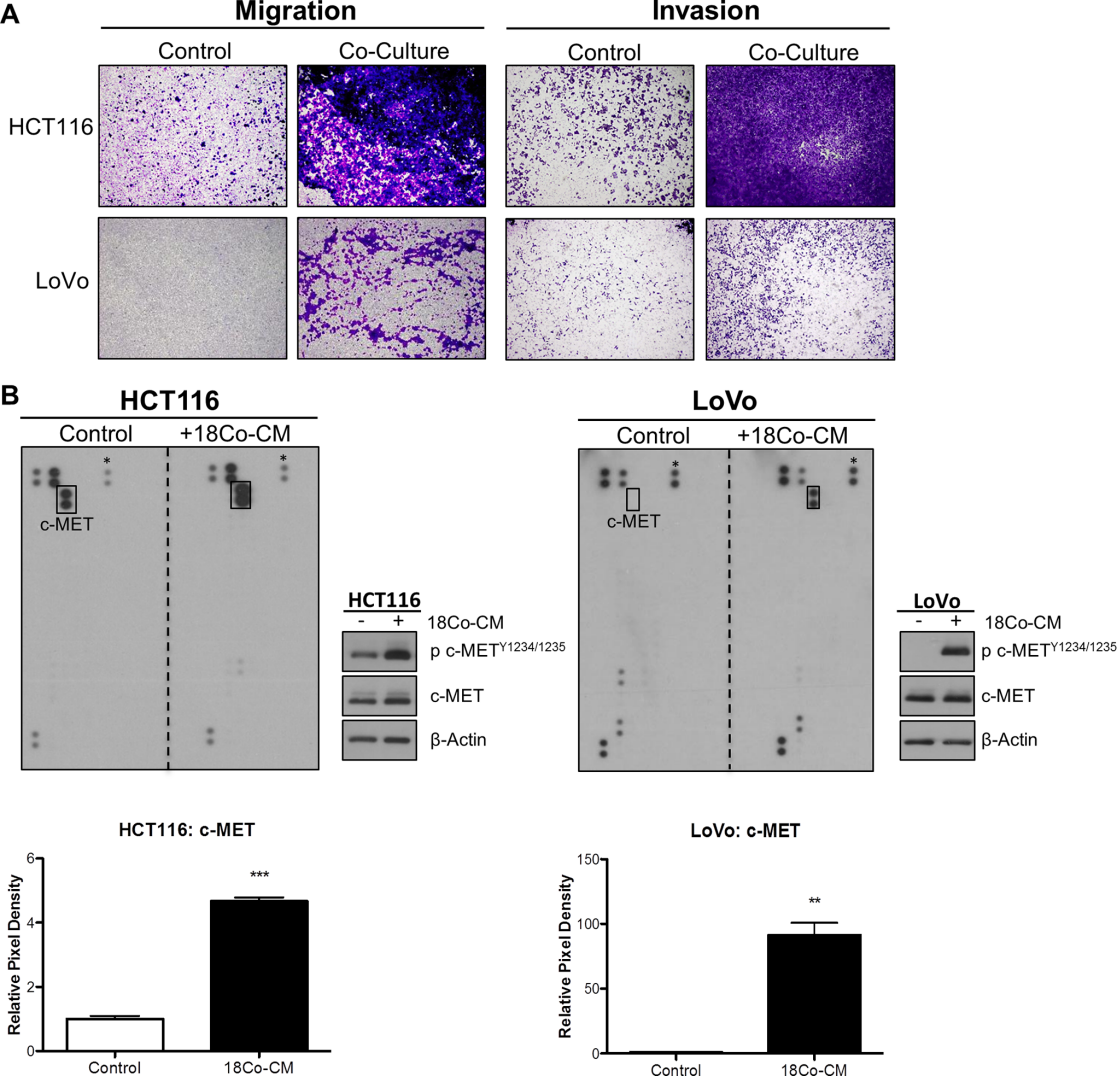
Myofibroblast-derived HGF promotes migration and invasion of CRC cells (**A**) HCT116 and LoVo cell lines were indirectly co-cultured with the colon fibroblast cell line CCD-18Co and the effect on both migration and invasion was determined using Boyden chambers. (**B**) Human phospho-receptor tyrosine kinase (pRTK) array in HCT116 and LoVo cells, cultured with or without conditioned media from CCD-18Co fibroblast cell line (18Co-CM). Total phosphorylated MET protein levels was further quantified by pixel density assessment and Western blotting. *= Reference for normalisation.

We assessed the role of exogenous HGF in promoting migration and invasion of CRC cells. In order to model the CRC tumor microenvironment (TME), we used the normal colonic myofibroblast cell line CCD-18Co to represent tumor-associated stromal fibroblasts. Initial phenotypic characterisation showed that HCT116 cells displayed a change from an epithelial to an elongated, spindle-shaped mesenchymal morphology 24h following incubation with conditioned medium derived from CCD-18Co cells (CCD-18CoCM) (Supplementary Figure S3A). Using an indirect co-culture system, we observed marked increased migration and invasion of HCT116 and LoVo cells following 48h co-culture with CCD-18Co cells (Figure 3A). Although incubation of CRC cells with CCD-18CoCM for 72h resulted in a 1.2-fold and 1.5-fold increased proliferation rates in HCT116 and LoVo cells respectively, the increased invasion rates of HCT116 and LoVo cells when co-cultured with CCD-18Co cells occurred already within the first 24h (Supplementary Figure S3B).

**Figure 4:**
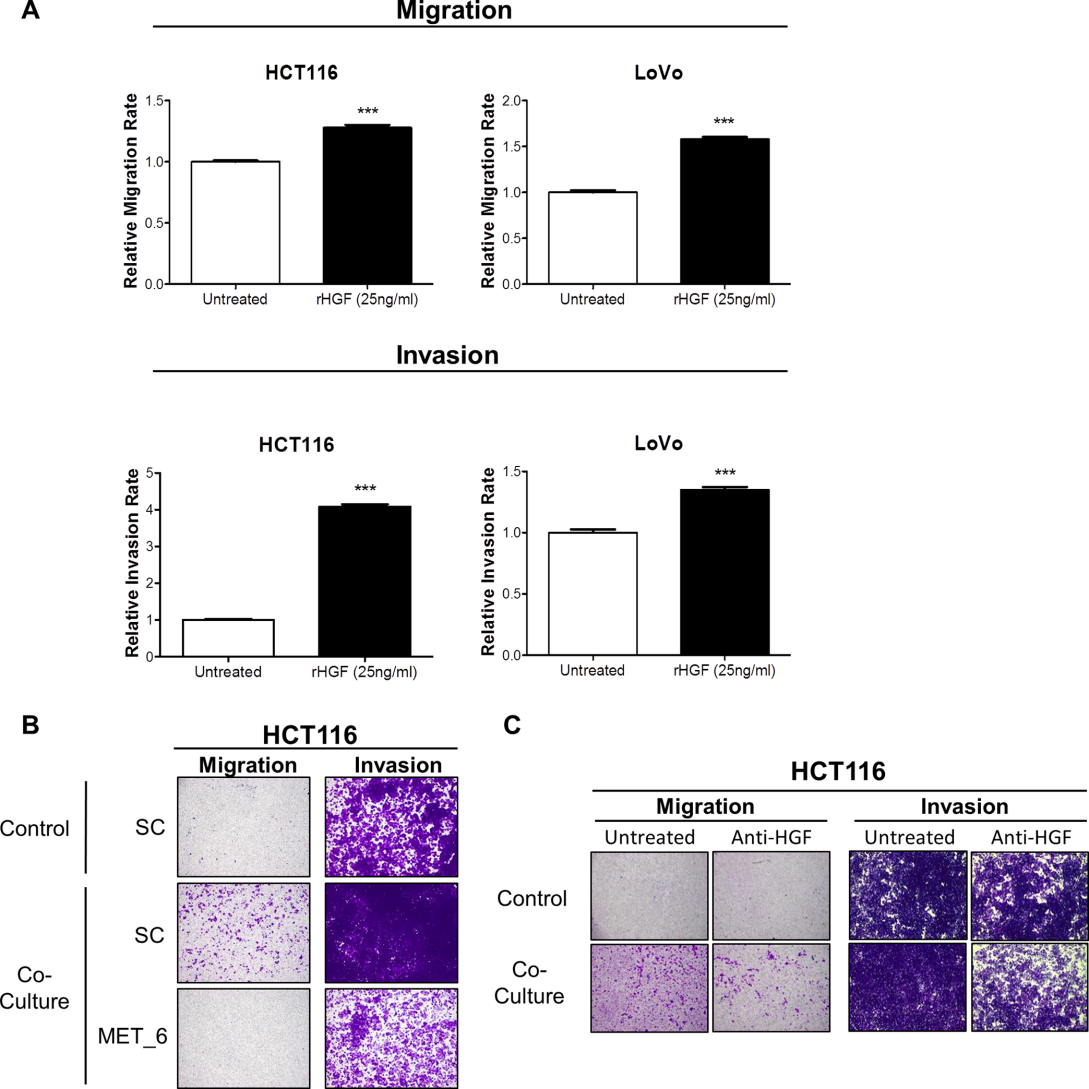
Recombinant HGF promotes migration and invasion of CRC cells (**A**) Migration and invasion rates of HCT116 and LoVo cell lines were measured using the xCELLigence system in the absence and presence of 25 ng/ml rh-HGF. (**B**) Boyden chamber assessment of HCT116 migration and invasion, following transfection with 10 nM SC or 10 nM siMET and indirect co-culture with CCD-18Co cells. (**C**) Boyden chamber assessment of HCT116 migration and invasion following co-culture with CCD-18Co cells, in the absence or presence of anti-HGF neutralising antibody. ***= *p* < 0.001.

In order to identify the signalling mechanism which drives increased migration/invasion in our CRC models following co-culture with CCD-18Co cells, we assessed the phosphorylation status of 42 RTKs using a human phospho-RTK array kit. The phospho-RTK array images and matched densitometry data showed significant increased c-MET tyrosine phosphorylation levels in HCT116 (4.67-fold; *p* < 0.001) and LoVo (91.39-fold; *p* < 0.01) cells following 15-minutes incubation with CCD-18CoCM compared to the unstimulated cells (Figure 3B). We validated our array results by Western blotting, using anti- Y^1234/1235^ c-MET and total c-MET antibodies. Phosphorylation of Y^1234/1235^ c-MET, but not total c-MET levels, was markedly increased in HCT116 and LoVo cells following 15 min incubation with CCD-18CoCM (Figure 3B). Further data showed significant increases in *HGF* mRNA and protein levels in CCD-18Co cells compared to levels measured in the HCT116 cells (Supplementary Figures S1 and S3C), indicating that myofibroblast-derived HGF plays a key role in c-MET activation and migration/invasion of CRC cells following co-culture with CCD-18Co cells. In addition, stimulation of HCT116 and LoVo cells with recombinant human HGF (rh-HGF) (25 ng/ml) resulted in significant increases in migration (*p* < 0.001) and invasion (*p* < 0.001) rates (Figure 4A), confirming the key role of the HGF/c-MET axis in mediating CRC cell migration and invasion in the context of the TME. siRNA-mediated knockdown of MET and neutralisation of HGF using an anti-HGF monoclonal antibody was sufficient to abrogate the increased migratory/invasive potential of CRC cells following co-culture with CCD-18Co cells (Figure 4B and 4C).

### Recombinant and myofibroblast-derived HGF promotes dynamic downregulation of c-MET protein levels

**Figure 5:**
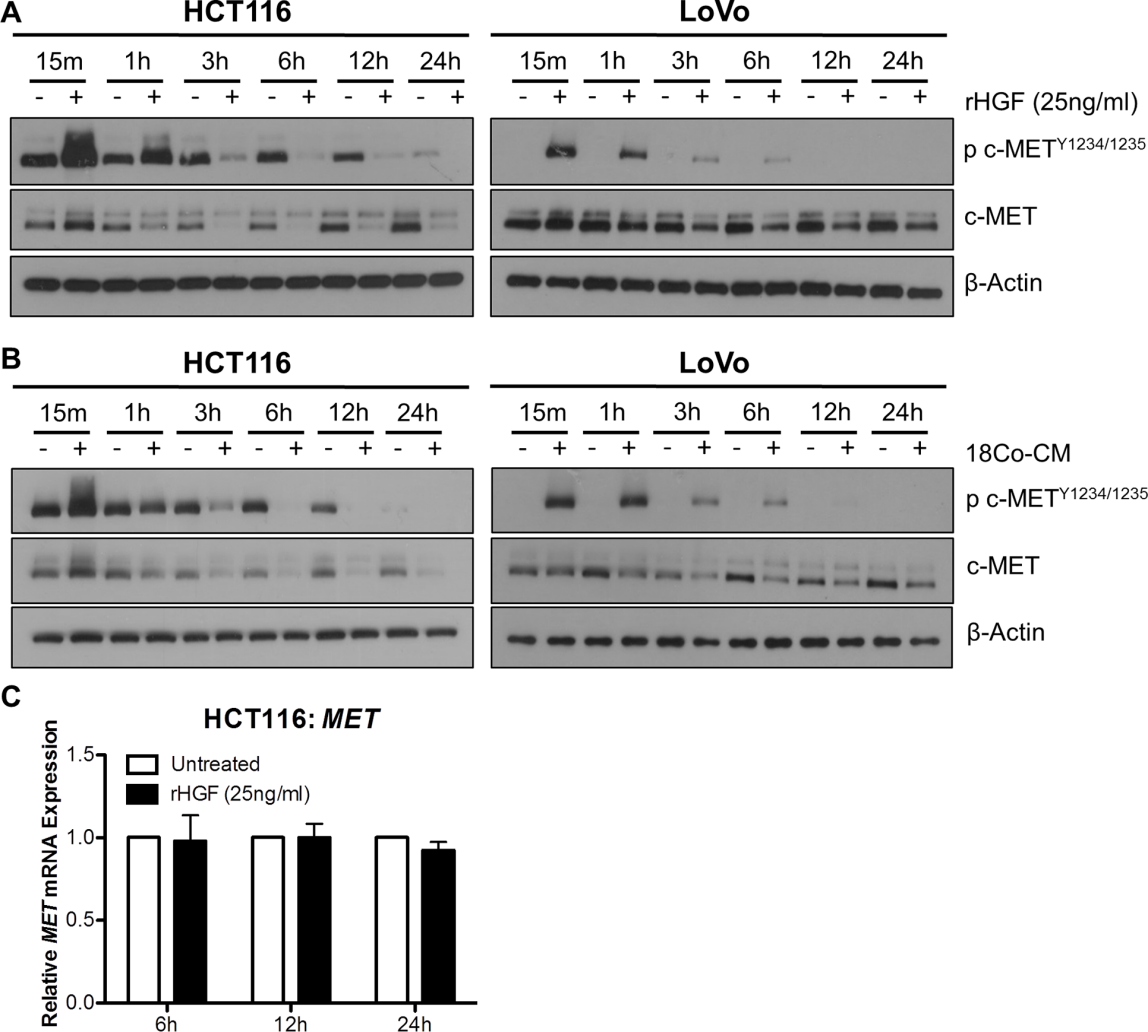
Exogenous HGF results in rapid downregulation of c-MET protein but not MET mRNA levels A + B. Western blot analysis of pMET^1234/1235^ and MET levels in HCT116 and LoVo cells following stimulation with rh-HGF (**A**) or incubation with conditioned media from CCD-18Co cells (18Co-CM) (**B**) for 15 minutes, 1, 3, 6, 12 and 24 hours. (**C**) qRT-PCR quantification of the corresponding *MET* gene expression levels at 6, 12 and 24 hours following culture in the absence or presence of rh-HGF.

In order to assess the effect of HGF stimulation on the stability of c-MET protein levels, we performed a time-course experiments with rh-HGF treatment in our CRC cells. Marked increases in c-MET^Y1234/1235^ levels were observed in HCT116 and LoVo cell lines, 15 min and 1 hour following stimulation with 25ng/ml rh-HGF; however at the latter time-point, downregulation of total c-MET levels was observed (Figure 5A). Similar results were seen following co-culture of HCT116 and LoVo cells with CCD-18Co fibroblasts (Figure 5B). In contrast to c-MET protein levels, *MET* mRNA levels remained unchanged, 6, 12 or 24 hours following stimulation with rh-HGF (Figure 5C). These data highlight that the unstable nature of c-MET protein levels is not reflected at the transcriptional level, and therefore that *MET* mRNA expression levels may be a more robust readout of biological overexpression and dependency on c-MET.

### *MET* transcription, but not protein expression, is upregulated in budding CRC cell populations in stage III primary CRC tumors

**Figure 6:**
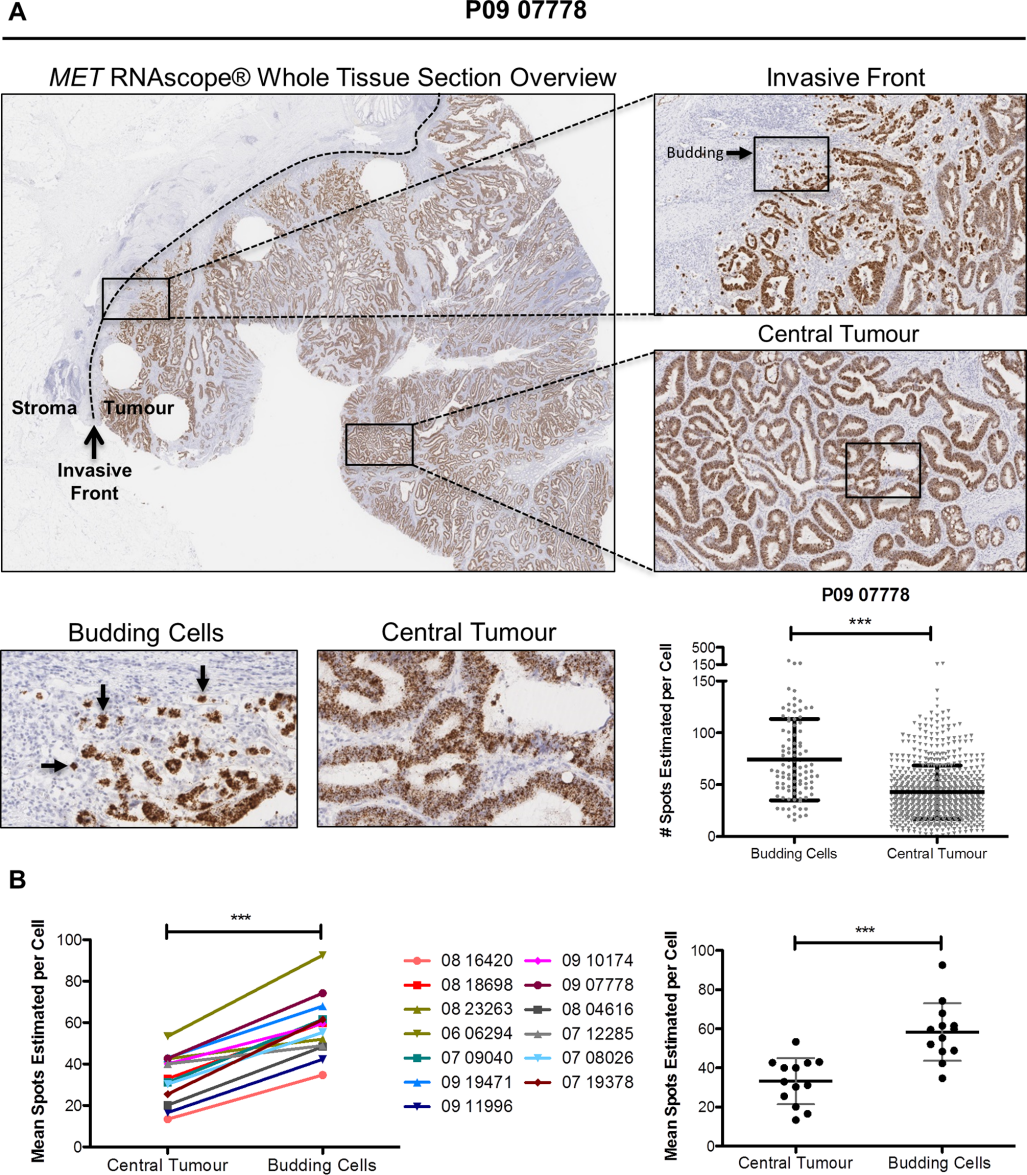
Budding cells at CRC invasive front harbour increased *MET* mRNA levels (**A**) Representative image of *MET* RNA *in situ* hybridization (RNA ISH) across a full-face tumor section at low magnification (x1 top left) and higher magnification for selected regions (x5, top right). Differential expression of *MET* mRNA between the central tumor and budding cells at the invasive front was observed qualitatively (x20, bottom left) and determined to be significantly different using SpotStudio software analysis (*p* < 0.001) (bottom right). (**B**) *MET* mRNA levels at multiple annotated regions of interests were determined using SpotStudio software across all 13 CRC cases. Mean *MET* mRNA levels at each region are indicated using paired assessment (left) and group analysis (right). *** = *p* < 0.001.

To support the results of our *in vitro* experiments, we examined *MET* gene and protein expression *in situ* in budding tumor cells at the invasive front using CRC clinical tissues. Budding tumor cells at the CRC invasive front are potentially exposed to high physiological concentrations of stromal-derived paracrine ligands through either direct or indirect contact. Using whole face sections of primary stage III CRC tumors (Table 1, n=13), we performed RNA *in situ* hybridisation (RNA ISH) using a probe specific for *MET* (Figure 6A). While there appeared to be only small fluctuations in transcription levels across the entire section, we performed detailed examination of multiple regions at the invasive front compared to the central tumor. Using a digital pathology method (See Materials and Methods) to measure the signal intensity, converting signal intensity into a digital surrogate measure of single cell transcript levels, we found a significant increase in *MET* transcription in budding cells compared to the central tumor regions (*p* < 0.001) (Figure 6A). While there was some variation in the baseline transcript levels in the central regions between tumors, we consistently observed an increase in the transcript levels in budding cells across all 13 tumor budding-positive tumors examined, regardless of the associated central region transcriptional levels within each tumor (*p* < 0.001) (Figure 6B). The cell-specific source of *MET* expression was confirmed by both molecular analysis and pathology-based assessment. Using Haematoxylin and Eosin (H&E) evaluation of the whole tissues sections and E-Cadherin staining, *MET* gene expression was localised to the epithelial cells (Supplementary Figure S4). In addition, bioinformatics assessment of microarray profiles obtained from dissociated fresh primary tumors (GSE39396), which had been fluorescence activated cell sorting (FACS) selected into specific endothelial, epithelial, leukocyte and fibroblast populations, confirmed that *MET* gene expression is significantly higher in tumor epithelial cells when compared to endothelial cells or leukocyte or fibroblast cells within the TME (*p* < 0.0001) (Supplementary Figure S5).

**Table 1:** Clinico-pathological features of the stage III CRC patient cohort

Patient characteristics	*N* = 13
Age (y)	
Median	64
Range	39–73
Gender	
Male	8 (62%)
Female	5 (38%)
T Stage	
pT0	0
pT1	0
pT2	0
pT3	6 (46%)
pT4a	0
pT4b	7 (54%)
N Stage	
N0	0
N1	9 (69%)
N2	4 (31%)
M Stage	
M0	13 (100%)
M1	0
Staging	
IIB	0
IIIA	1 (8%)
IIIB	8 (61%)
IIIC	4 (31%)
IV	0
Tumor Type	
Adenocarcinoma	12 (92%)
Mucinous Carcinoma	1 (8%)
Differentiation	
Well - Moderate	11 (77%)
Poorly	3 (23%)
Tumor Site	
Caecum	5 (39%)
Ascending Colon	2 (15%)
Transverse Colon	0
Sigmoid Colon	3 (23%)
Splenic Flexure	1 (8%)
Rectum	2 (15%)
Lymphovascular Invasion (LVI)	
Yes	10 (76%)
No	3 (24%)
Extramural Venous Invasion (EMVI)	
Yes	4 (31%)
No	9 (69%)
Perineural Invasion (PI)	
Yes	0
No	8 (62%)
Uncertain	5 (38%)
Tumor Budding	
Yes	13 (100%)
No	0

As our *in vitro* models showed instability of c-MET protein following co-culture with CCD-18Co cells and stimulation with rh-HGF, we assessed c-MET protein levels in our clinical tissues. IHC analysis revealed that although differences in basal c-MET protein levels could be detected between individual tumors, no increased c-MET protein levels were observed between the central and budding cells in individual tumors (Supplementary Figure S6).

## DISCUSSION

The establishment of metastasis following the development of a primary tumor, requires a number of well-defined steps, however the molecular factors associated with metastatic progression still remain poorly understood [29]. Our previous data and other studies have identified a role for AXL in cancer cell migration/invasion and showed that AXL levels are required for niche activation and the first phase of metastatic colonization [21, 30]. However, delineation of the key molecular drivers of the initial invasive event in CRC tissues requires a detailed investigation of single budding cells at the invasive front, which will open the way for novel and tailored approaches to target metastatic spread and increase outcome of patients with early stage CRC.

Using unsupervised classification of gene expression profiles, a number of research groups have published 3–6 molecular subtypes within stage II/III CRC [18, 19, 31–33]. Recent consensus molecular subtype analysis by the CRC Subtyping Consortium (CRCSC) has coalesced these independent classification systems into 4 Consensus Molecular Subtypes (CMS 1–4) [6]. Of these subgroups, the mesenchymal subtype (CMS4) has been associated with poor differentiation and the poorest patient outcome. Although the initial studies suggested that cancer cells of epithelial origin within CMS 4 had acquired mesenchymal traits and stem cell-like properties, resulting in increased invasion and metastatic spread, more recent studies have shown that the distinctive gene expression profiles and clinical features of CMS4 are due to their profuse stromal cell component [7, 8]. While the latter studies argue against a widespread EMT across the entire tumor, these studies do not exclude the possibility that individual tumor cells may undergo EMT, particularly at the invasion front and in budding cells. Indeed, previous studies have shown that tumor cells at the invasive front and budding cells have a strong nuclear staining pattern for b-catenin, which is associated with loss of membranous E-cadherin and Ki-67 expression, epithelial and proliferation markers respectively [34, 35]. Not surprisingly, high-grade tumor budding in CRC has been associated with poor patient outcome, pathological invasion, and metastasis to the lymph node and liver [4, 5]. Based on the findings from our *in vitro* models, we performed focused single cell profiling of individual CRC tumor buds, in order to reveal the precise biology associated with the invasive capacity of these budding cells.

Oncogenic deregulation of the HGF/c-MET pathway has been reported in a wide range of human cancers, including breast [36], ovarian [37], CRC [25], prostate [38], pancreatic [39], gastric [40, 41] and non-small cell lung carcinoma [42], where it is often associated with drug resistance, enhanced migration/invasion, metastasis and a poor clinical outcome [41, 43, 44]. The apparent overexpression of *MET* at mRNA and protein levels, using qRT-PCR, microarray transcriptional profiling and IHC, has been reported by a number of independent groups in CRC [24, 26, 45–48], with a recent meta-analysis of a number of these studies offering the consensus that overexpression of c-MET and HGF occurs at a frequency of up to ~78% and ~95% respectively [49]. In addition, elevated c-MET expression and/or amplification has also been associated with depth of tumor (T) invasion, lymphovascular invasion, presence of regional lymph node and distant metastatic disease in CRC [24–26, 50].

In order to model CRC cell invasion, our group has previously developed invasive CRC daughter cells which displayed an EMT-like phenotype and high levels of CD44 [21]. We now show that c-MET protein and mRNA levels are highly expressed in these models and in our newly developed invasive CRC daughter cell lines and that siRNA mediated knockdown of c-MET potently inhibited migration and invasion of parental and invasive CRC cells. We next demonstrated that myofibroblast-derived conditioned medium can further potentiate migration and invasion of CRC cells through HGF-dependent activation of c-MET in CRC cells. Importantly, we found that RNAi against c-MET and monoclonal antibodies against HGF abrogated the increased migratory and/or invasion potential following co-culture of CRC cells with colon myofibroblasts. Taken together, these studies suggest a key role for HGF-independent and HGF-dependent activation of c-MET as a molecular driver of CRC cell migration/invasion *in vitro*.

To underpin the results of our *in vitro* experiments, we used a novel RNA ISH approach for single cell analysis of tumor budding foci in a cohort of stage III CRC tumor samples. These data showed marked upregulated *MET* mRNA expression in budding tumor cells, compared to levels obtained in tumor cells within the central tumor, regardless of the overall *MET* mRNA levels in the central regions. Although the CRC cases selected for this study most likely have different mutational spectra, the increased *MET* gene expression levels in tumor budding foci were consistently observed in every CRC tumor tissue sample analysed. This finding confirms the homogeneous nature of *MET* gene expression association with actively invading cells and tumor buds. Importantly, this transcriptional event was not reflected at the protein level, as measured by IHC. Previous studies, including our own data, have shown that growth factor binding to plasma membrane receptor tyrosine kinases, such as the Epidermal Growth Factor Receptor (EGFR), EpHA2 and c-MET result in rapid internalization and degradation of these receptors, providing a mechanism for preventing sustained and uncontrolled pathway activation [20, 51]. A number of studies, including our current data, have shown that c-MET and HGF are detected primarily in epithelial cancer cells and stromal cells respectively [52]. These data support our hypothesis that the discordance between c-MET protein and *MET* gene expression, detected in tumor budding cells, is a consequence of HGF-dependent internalisation and degradation of the c-MET protein, as budding tumor cells are likely to be exposed to high physiological concentrations of stromal-derived HGF. Thus, the widespread adoption of IHC for biomarker analysis is clearly not optimal for proteins such as c-MET, which undergo rapid internalisation and degradation after exposure to stromal-derived factors.

Previous studies carried out to assess a potential prognostic role for c-MET in CRC have shown inconsistent results. These discrepant published data reflect not only the differences in technical detection methodologies used (IHC, qRT-PCR, FISH) but also the wide variety and subjective scoring criteria used for IHC [26, 53–57]. As *MET* gene expression is not subject to ligand-dependent negative regulation (Figure 5), our study also indicates that the use of a transcriptional semi-quantitative method such as RNA ISH may be a more appropriate technology for measuring the prognostic significance of c-MET in invasive CRC. A limitation of our study was the small sample size of our pilot study, and a larger prospective study is planned to assess c-MET expression in stage II/III CRC, using both *MET* RNA ISH and IHC analysis of tumor budding foci, invasive front and central tumor and correlate with patient outcome. In addition, the ongoing phase I/II MErCuRIC clinical trial of combined c-MET/MEK1/2 inhibition in *RAS*MT and *RAS*WT metastatic CRC with aberrant c-MET expression, is assessing *MET* RNA ISH, IHC and DDISH in CRC liver metastasis and its potential as predictive biomarker [58].

The use of a single cell profiling method, such as RNA ISH, has allowed us to begin to uncover the transcriptional signalling that is associated with early invasive CRC. In addition, the cell-specific precision of this approach enables accurate delineation of the source of the detected transcript. RNA ISH also allows a cell-by-cell dissection of the detailed biology underpinning tumor progression in CRC, which is not routinely possible using standard transcriptomics approaches.

In conclusion, using preclinical CRC models and patient tissue samples, we have identified c-MET as a key regulator of CRC cell migration and invasion. A variety of therapeutic strategies have been developed to target the c-MET axis, including monoclonal antibodies and both selective and unselective tyrosine kinase inhibitors. Our data provide support for the further investigation of c-MET as a target in early stage III CRC patients who exhibit high *MET* mRNA levels in budding cells and the invasive front.

## MATERIALS AND METHODS

### Cell culture

Acquisition, authentication, and culture of HCT116, HKH-2, LoVo, and DLD-1 cells has previously been described [21]. CCD-18Co cells were purchased from American Type Tissue Culture Collection (ATCC), and cultured in Eagle's Minimum Essential Medium (EMEM) according to the manufacturers recommendations. All cell lines were authenticated (by short tandem repeat (STR)- profiling and karyotyping).

### Selection of invasive CRC subpopulations

BD BioCoat Matrigel Invasion Chambers (BD Biosciences) were used to isolate invasive cell subpopulations from CRC cell lines, as described previously [21]. In brief, CRC cells were seeded in serum-free Dulbecco's Modified Eagle Medium (DMEM) into the upper chamber, and allowed to invade for 72–96 hours towards chemoattractant (10% serum DMEM) in the lower chamber. Invaded cell populations were subsequently sub-cultured, and termed “invasive”.

### *In vitro* migration and invasion assays

Real-time migration and invasion were assessed in real-time using the CIM-plate 16 and the xCELLigence system (Roche Applied Sciences, City Country) according to the manufacturer's instructions, and as previously described [21]. For classical migration/invasion assays, Corning Transwell PET migration Chambers (Corning) and BD BioCoat Matrigel Invasion Chambers (BD Biosciences) were used as described previously [20, 21].

### siRNA transfections, flow cytometry, western blotting, qRT-PCR, ELISA and RTK array profiling

Transfections, Flow Cytometry, and Western blot analysis have been previously described [21]. Real-time quantitative PCR (qRT-PCR) was performed as previously described [20, 21]. The Proteome Profiler Human XL Cytokine Array Kit (R&D Systems) was used for pRTK array profiling, and ImageJ software (Fiji) for quantification. The RayBio^®^ Human HGF ELISA kit was used for quantification of HGF secretion, as previously described [27].

### Patients and samples for the pilot study

For assessment of c-MET expression at the invasive edge and in the central tumor, FFPE primary CRC tumor blocks from 99 patients who received adjuvant XELOX treatment were obtained (NIB12–0034). From this cohort, H&E stained serial whole tissue sections were microscopically examined and 13 cases were identified having i) a clear central tumor region, incorporating neoplastic glands distal from the stroma; ii) an invasive front, with a clear tumor-stroma boundary; iii) a surrounding stromal region adjacent to the invasive edge; iv) evidence of tumor budding.

### RNAscope staining and quantification of MET mRNA expression

Sections from the selected 13 FFPE tumor blocks were stained for *MET* mRNA using the RNAscope^®^ 2.0 HD Detection Kit (Brown) for FFPE Tissues (Advanced Cell Diagnostics, Hayward, CA; #310035). Briefly, sections were cut at 4 μm, air-dried overnight, baked at 60°C for 1 hour, dewaxed, and air-dried before pre-treatments. For all probes, a standard pre-treatment protocol was used. Three RNAscope^®^ probes were employed in this study: Homo sapiens MET (Hs-MET) (Cat. No. 423101 - sequence region 175–6505), positive control probe Homo sapiens ubiquitin C (Hs-UBC) (Cat. No. 310041 – sequence region 342–1503), and negative control probe dihydrodipicolinate reductase (bacterial *dapB*) (Cat. No. 310043 – sequence region 139–989). Slides were scanned using an Aperio scanner at 40X resolution. Quantification of *MET* mRNA in selected regions of interest (ROI) was performed using SpotStudio™ software (Definiens and Advanced Cell Diagnostics). ROI's included i) budding cells at the leading edge of the tumor, defined as small clusters of five or less cells located in the stroma, which have broken away from the epithelial glands at the invasive edge, and ii) central tumor regions, defined as epithelial cells with glandular morphology located distal to the invasive edge. Results for these selected cases were expressed as mean number of spots estimated per cell.

### c-MET immunohistochemistry and scoring

For IHC evaluation of c-MET protein expression, the CONFIRM Anti-Total c-MET (SP44) Rabbit Monoclonal Primary Antibody (Ventana^®^, Cat. No. 790–4430) was used. Sections for IHC were cut at 4 μm on a rotary microtome, dried at 37°C overnight, and then used for IHC, which was performed on an automated immunostainer (Leica Bond-Max, Milton Keynes, UK). A validated and optimized in-house protocol was used for c-MET IHC. Antigen-binding sites were detected with a polymer-based detection system (Bond, Newcastle Upon Tyne, UK; cat. no. DS9800). All sections were visualized with diaminobenzidine, counterstained with hematoxylin, and mounted in DPX.

Staining reaction for c-MET IHC was graded on a 4 four-tiered system: 0: no staining or < 10% of tumor cells stained at weak intensity, 1+: weak staining in > 10% of the tumor cells in the region of interest (ROI), 2+: moderate staining in > 10% of the tumor cells in the ROI, 3+: strong staining in > 10% of the tumor cells in the ROI. Only cytoplasmic and membranous expression was evaluated.

### Gene expression dataset

Gene expression profiles from an independent CRC dataset were downloaded from NCBI Gene Expression Omnibus (GEO) (http://www.ncbi.nlm.nih.gov/geo/) under accession number GSE39396. GSE39396 contains microarray profiles from fresh colorectal specimens where Fluorescence Activated Cell Sorting (FACS) selected cells into specific endothelial [CD45(+), EPCAM(–), CD31(–), FAP(–)], epithelial [CD45(–) EPCAM(+), CD31(–), FAP(–)], leukocyte [CD45(–), EPCAM(–), CD31(+),FAP(–)] and fibroblast [CD45(–), EPCAM(–), CD31(–), FAP(+)] populations.

### Transcriptional analysis

Partek Genomics Suite^®^ 6.6 software (Partek Inc.) was used for dataset analysis. The data was uploaded and underwent RMA normalisation prior to downstream analysis. Expression values for the 4 probesets representing *MET* were selected (203510_PM_at; 211599_PM_x_at; 213807_PM_x_at; 213816_PM_s_at). For the purpose of clustering, the data matrices were standardized to the median value of probeset expression. Following standardization, 2-dimensional hierarchical clustering was performed (samples x probe sets/genes). Hierarchical clustering was carried out using Euclidean distance with Ward's linkage method.

### Statistical analyses

GraphPad Prism 5 Software (Graphpad.com) was used for all statistical tests of *in vitro* data. Relative expression box and whisker data was plotted as median probeset values (10% and 90% values indicated by whiskers). Additionally, relative qRT-PCR expression, cell cycle values and migration/invasion rates were also plotted. Unpaired Students *t*-tests were used to determine significance (*p* < 0.05) of indicated groups. ANOVA tests were used to determine statistical significance of multiple groups, with Tukey's post hoc correction.

## SUPPLEMENTARY MATERIALS


