# Supplementary material for: Transcriptional upregulation of c-MET is associated with invasion and tumor budding in colorectal cancer
Source: Oncotarget. 2016 Oct 26;7(48):78932–45. doi: 10.18632/oncotarget.12933 (PMC5346688; doi:10.18632/oncotarget.12933)
Supplement: Supplementary file 1 [file oncotarget-07-78932-s001.pdf]

Transcriptional upregulation of c-MET is associated with invasion and tumour budding in colorectal cancer

Supplementary Materials

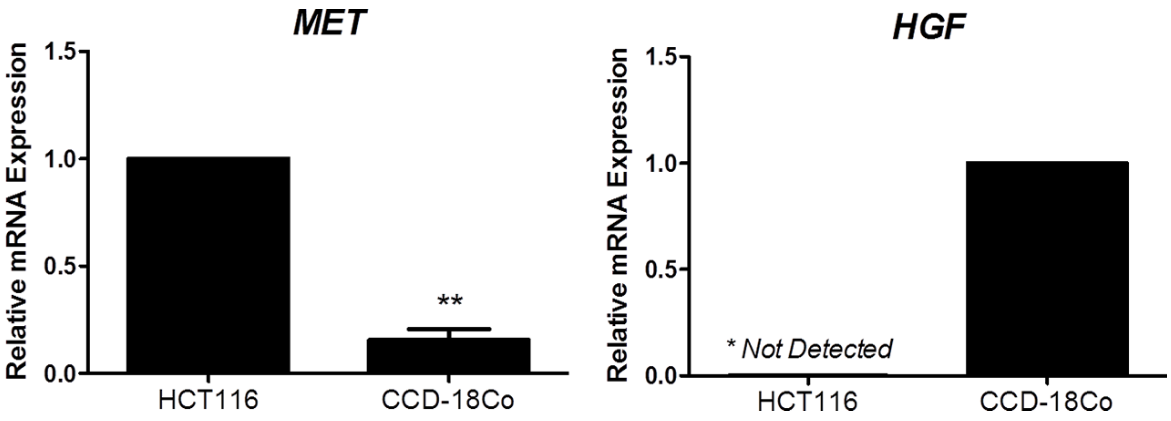

Supplementary Figure S1: *MET* and *HGF* mRNA levels in HCT116 CRC cells and CCD-18Co colon fibroblast cells. Q-PCR analysis of *MET* and *HGF* mRNA levels. Relative mRNA expression was calculated using the DDCt method with normalisation to GAPDH. ** = $p < 0.01$

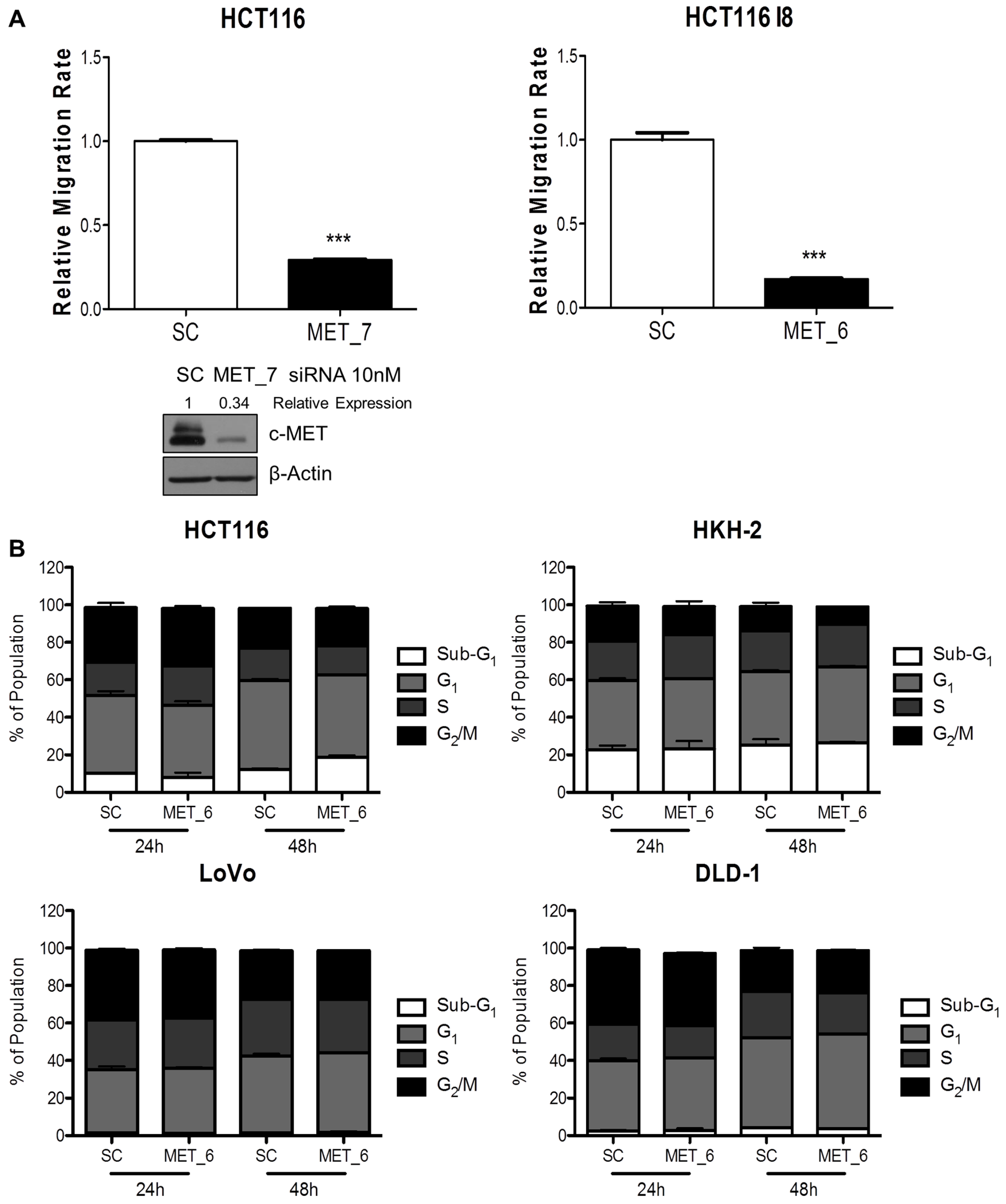

Supplementary Figure S2: Effect of RNAi against *MET* on migration, apoptosis and cell cycle distribution in parental and invasive CRC cells. (**A**) Parental and invasive (I8) HCT116 CRC cells were transfected with 10nM SC or 10nM *MET* siRNA (MET_6 or MET_7) for 24 hours and migration rates determined using the xCELLigence system and the CIM plate 16 (Roche Applied Sciences). c-MET expression levels were detected by Western blotting. (**B**) HCT116, HKH-2, LoVo and DLD-1 cells were transfected with scrambled control (SC) or c-MET specific (MET_6) siRNA for the indicated time and cell cycle profiles were assessed by flow cytometric analysis with PI.

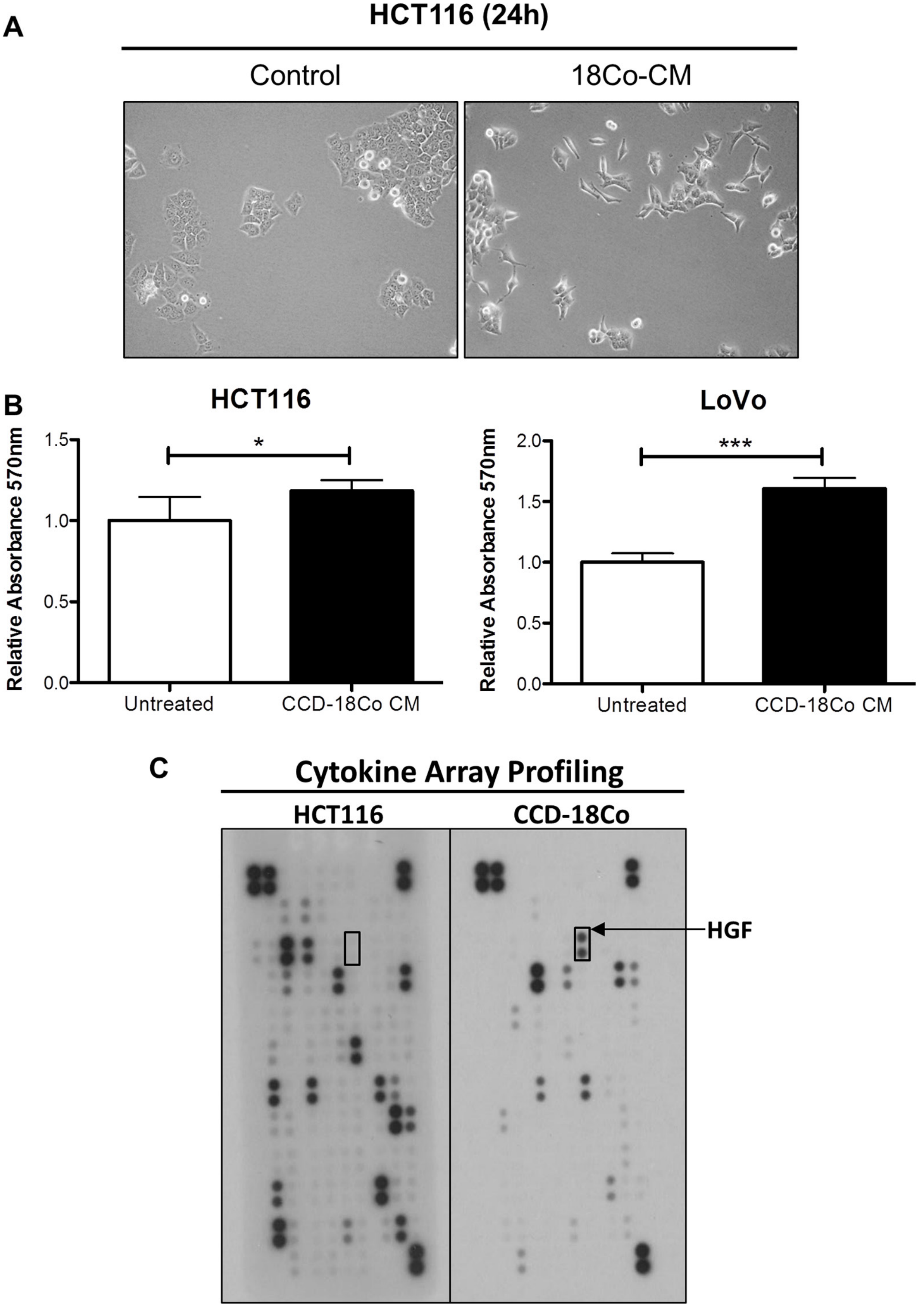

Supplementary Figure S3: Effect of the CCD-18Co secretome on cell morphology and proliferation of CRC cells. (**A**) Images of morphology of HCT116 cells following 24 hours incubation with conditioned medium of CCD-18Co fibroblasts. (**B**) Relative absorbance values of crystal violet stained cells following 72h culture of HCT116 and LoVo cells in conditioned medium of CCD-18Co fibroblasts. (**C**) Cytokine array using conditioned medium from HCT116 cells and CCD-18Co fibroblasts.

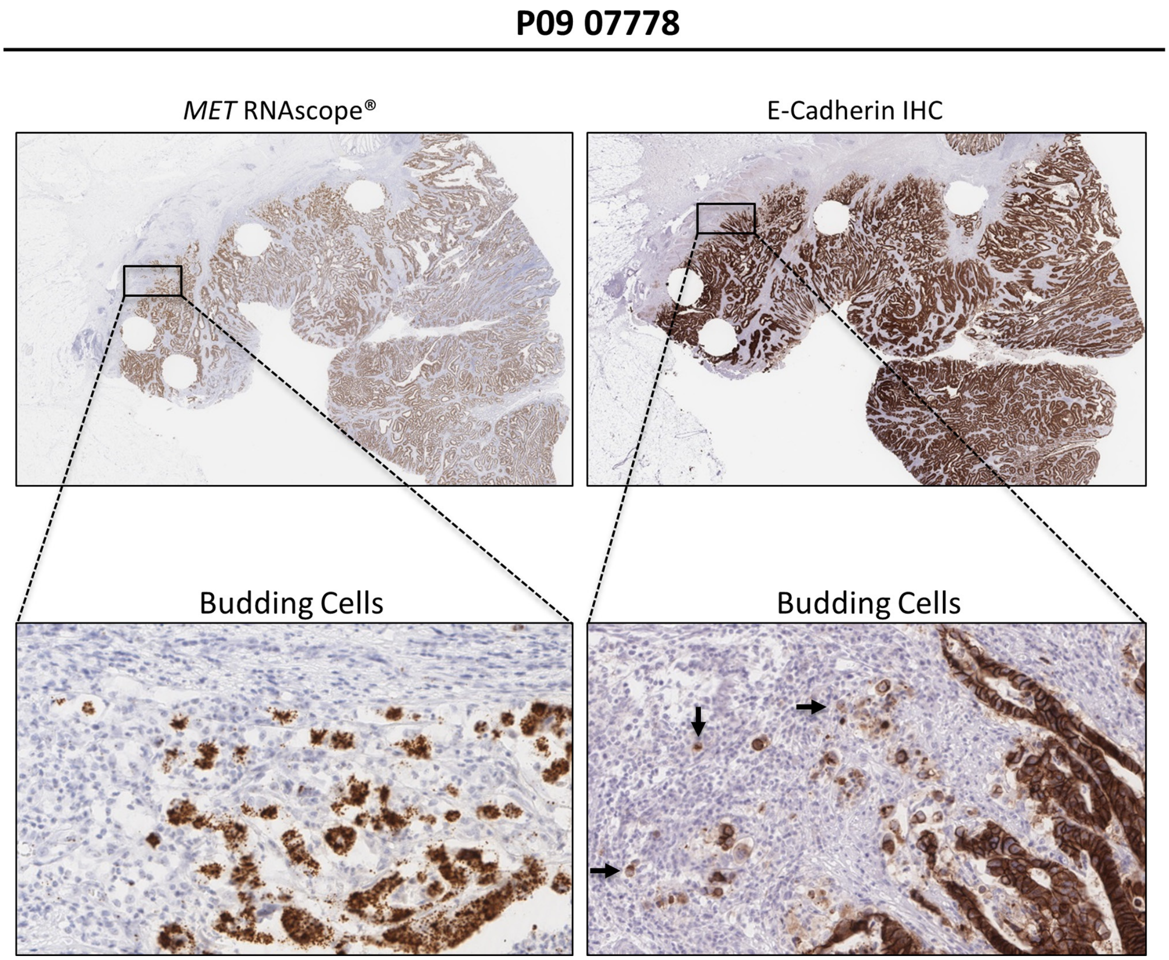

Supplementary Figure S4: Expression of E-Cadherin by IHC at the CRC invasive front. Serial sections of CRC tissue indicating simultaneous increases in *MET* transcription and loss of E-Cadherin protein expression in budding cells (arrows) compared to the tumor bulk.

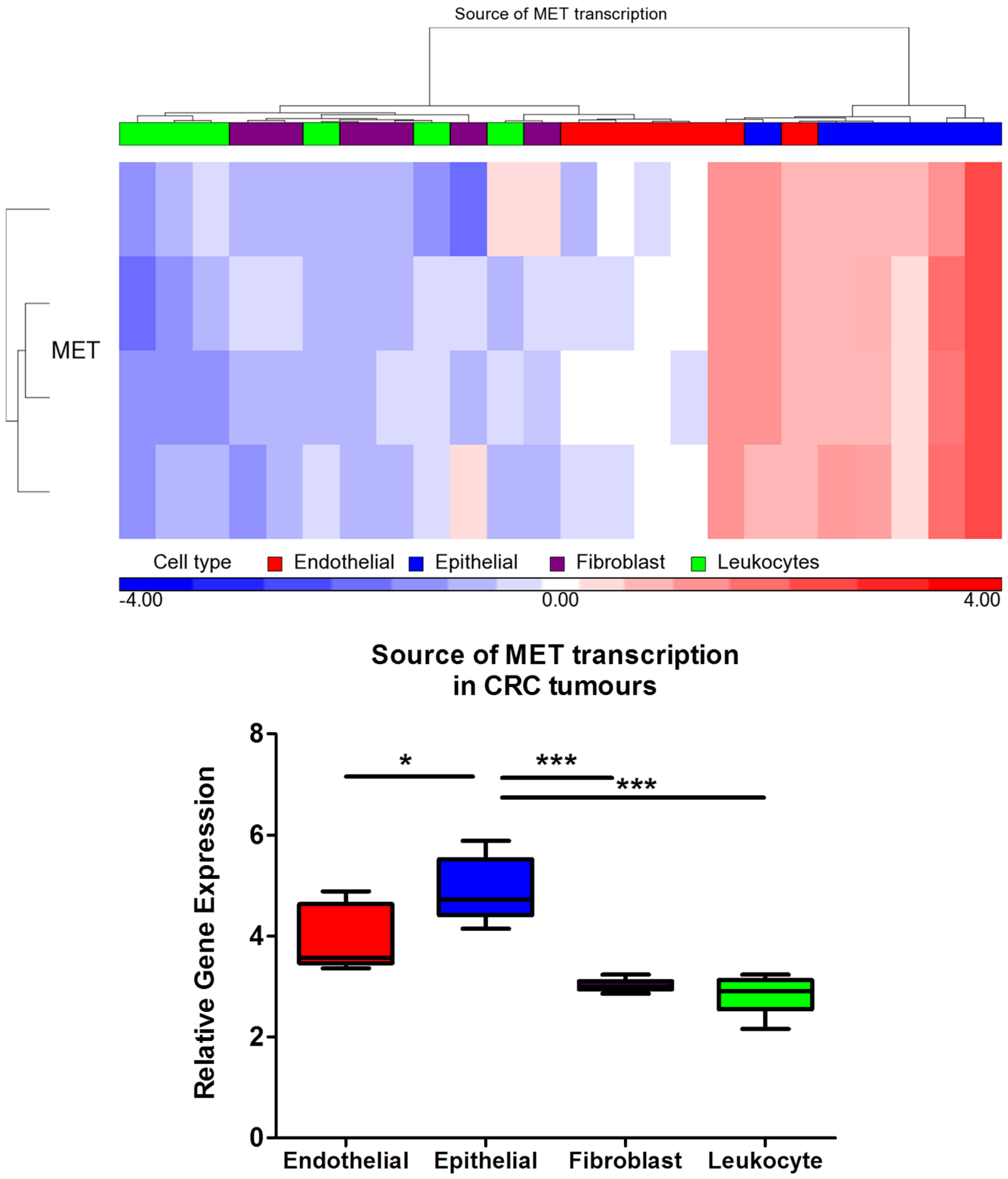

Supplementary Figure S5: Gene expression levels of *MET* according to cell type in CRC tumours. Heatmap representation (top) and box and whisker plot (bottom) of *MET* gene expression according to specific endothelial, epithelial, fibroblast or leukocyte cell-of-origin from FACS sorted primary colorectal tumours. Significance values are indicative of a Tukey's post hoc test * = $p < 0.05$, *** = $p < 0.001$.

P09 07778

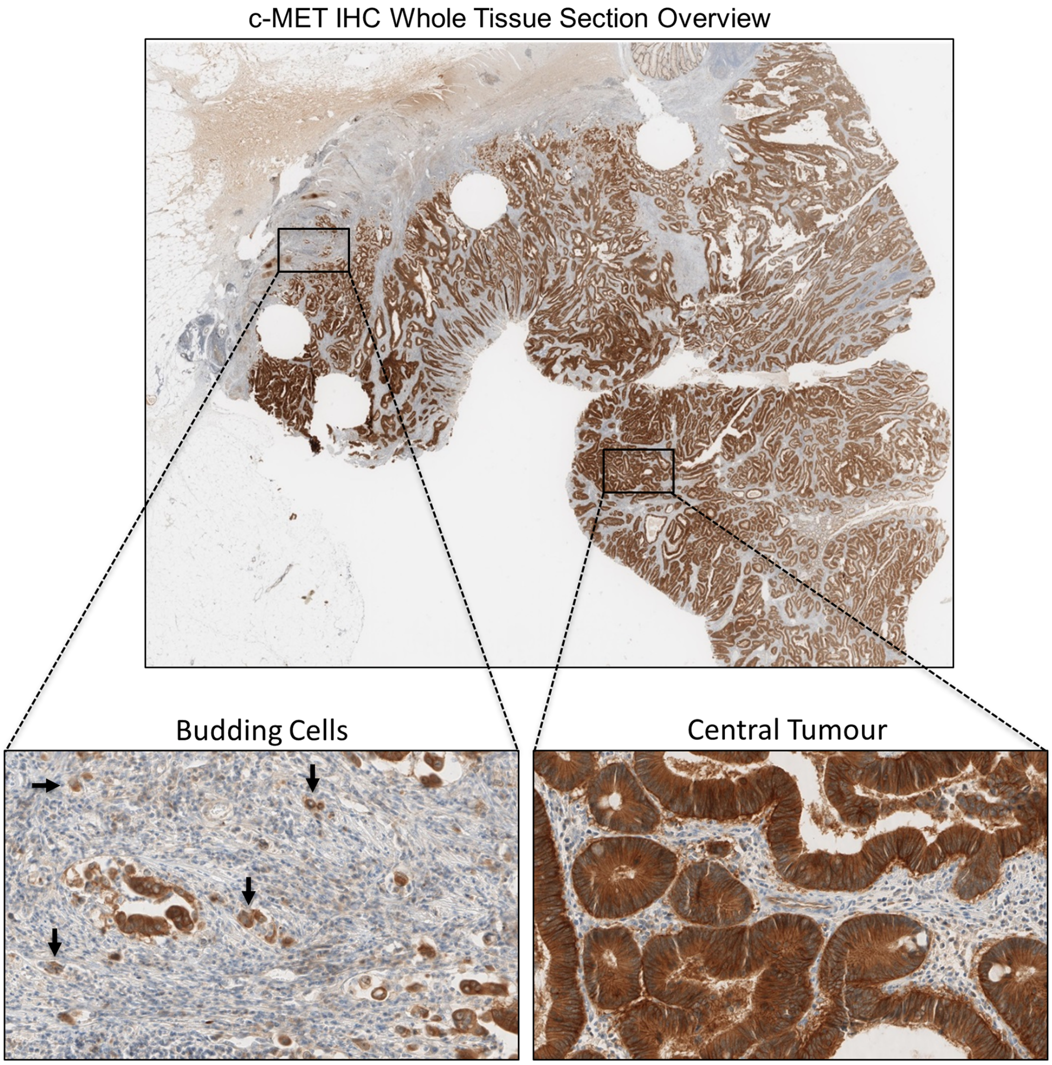

Path Number	Central Tumour	Budding Cells
06 06294	3+	3+
07 08026	3+	3+
07 09040	3+	3+
07 12285	2+	2+
07 19378	3+	3+
08 04616	1+	1+
08 16420	2+	2+
08 18598	3+	3+
08 23263	3+	3+
09 07778	3+	3+
09 10174	2+	2+
09 11996	3+	3+
09 19471	3+	3+

Supplementary Figure S6: Expression of c-MET protein using IHC at the central tumour and in budding cells at the invasive front. Semi-quantitative scoring was performed as described in Material and Methods with paired scoring indicated in table and plot.
